# Is oxytocin a trust hormone? Salivary oxytocin is associated with caution but not with general trust

**DOI:** 10.1371/journal.pone.0267988

**Published:** 2022-05-06

**Authors:** Qiulu Shou, Junko Yamada, Kuniyuki Nishina, Masahiro Matsunaga, Toko Kiyonari, Haruto Takagishi

**Affiliations:** 1 Tamagawa University, Tokyo, Japan; 2 Osaka University, Suita, Osaka, Japan; 3 Aichi Medical University, Nagakute, Aichi, Japan; 4 Aoyama Gakuin University, Sagamihara, Japan; Hamamatsu University School of Medicine, JAPAN

## Abstract

Studies on the association between trust and oxytocin, a neuropeptide of the central nervous system, have not reached a consensus, thereby challenging the possibility of a direct association between the two. However, previous studies have not examined how oxytocin is correlated with trust, based on its categorization into different factors in the field of social science. For instance, based on Yamagishi’s trust theory, trust can be categorized into two factors: general trust and caution. General trust refers to beliefs about the trustworthiness of others, whereas caution refers to the belief that caution is needed when dealing with high social uncertainty. In this study, to examine the relationship between these two factors and oxytocin, we analyzed data of 197 adults (men = 98, women = 99; mean age = 41.7 years; standard deviation for age = 10.4 years) and examined the relationships between these two factors of trust and endogenous salivary oxytocin levels. We found that oxytocin was positively correlated with caution rather than with general trust thereby suggesting that oxytocin plays a role in regulating caution rather than general trust among the components of trust. The present study demonstrated that salivary oxytocin level can act as a biomarker that partially predicts one’s trust, especially as reflected by caution.

## Introduction

Trust is a form of social capital and an indicator of a society’s maturity [1]. It has a role as a lubricant in interpersonal relationships and facilitates economic transactions in societies [2,3]. Growing bodies of research investigating the biological basis of trust, found that oxytocin regulates trust [4–6]. Oxytocin, a neuropeptide, is synthesized in the hypothalamus and acts as a neuromodulator in the central nervous [7]. Previous studies have shown that exogenous oxytocin promotes trust [4,5] and endogenous oxytocin (e.g., plasma oxytocin) and trust are associated [6]. However, recent studies [7,8] have questioned oxytocin’s function as a “trust hormone.” Nave et al. [9] pointed out that six previous studies attempted to replicate the study by Kosfeld et al. [5] and concluded that oxytocin administration could not promote trust. Moreover, Declerck et al. [8] failed to replicate the findings of a previous study by Kosfeld et al. [5]. Regarding endogenous oxytocin, some studies [10,11] found no association between plasma oxytocin levels and trust. Taken together, based on exogenous and endogenous studies, oxytocin may not be directly associated with trust.

The results of animal experiments can be helpful in understanding the function of oxytocin in living organisms. Based on the findings of animal studies [12–14], oxytocin is said to exert a central anxiolytic-like effect on endocrine systems and may have an important role in reducing anxiety-like behavior. For example, central oxytocin administration in rats can reduce stress-induced corticosterone release and reduce the anxiety-related behavior response to unfamiliar and complex environments [12]. In particular, Han et al. [15] suggested that the injection of oxytocin into the amygdala could reduce isolation-induced depression and anxiety-related behaviors. Consistent with this evidence, functional magnetic resonance imaging studies in humans revealed that the intranasal administration of oxytocin attenuated activity in the amygdala, especially during responses to fearful and angry faces [16] and the risk of betrayal in social interaction [4]. Moreover, social stress can induce oxytocin release to reduce anxiety in humans [17,18]. Based on these findings, researchers have proposed the anxiety reduction hypothesis to explain the effect of oxytocin on human sociality [19].

If oxytocin regulates anxiety, how does it relate to trust? Yamagishi’s emancipation theory of trust claims that trust consists of two independent factors [20,21]. One factor is general trust, a belief based on the expectation about the trustworthiness of others in general. The other factor is caution, the belief that caution is needed in situations of high social uncertainty. Increased levels of caution are associated with high perceived social risk and anxiety resulting from speculating about social deception or betrayal. The emancipation theory of trust emphasizes that the existence of caution is important for people with a high level of general trust to deal with social risk (e.g., judging the trustworthiness of a specific person) and to determine the level of trust in social interaction. In short, trust that appears as a behavior depends on two factors: general trust and caution.

These two factors are distinguished in the social sciences and have been discussed in various studies [20,21]. However, these important aspects of trust have been ignored in studies that investigated the relationship between oxytocin and trust. Considering the inconsistent results in the research addressing the oxytocin effect on promoting trust, oxytocin is possibly associated only with caution and not general trust; therefore, oxytocin sometimes does not accurately predict trust. In previous studies, such as the one by Baumgartner et al. [4], trust was measured by using an economic game called “the trust game.” Therefore, distinguishing between the two factors of trust was not possible. Based on the anxiolytic effect of oxytocin in animals and humans, oxytocin is capable of providing a sense of assurance and reducing the caution level when dealing with others. Israel et al. [22] found that oxytocin administration could decrease accuracy in the perception of social deception in humans, whereas it could not alter the perception of kindness in players of the prisoner’s dilemma game. These results imply that oxytocin cannot influence the perception of kindness in other people, whereas it can decrease caution toward social risk. Therefore, we hypothesized that oxytocin would be associated with caution but not with general trust.

## Materials and methods

### Ethics statement

Before the experiment, all participants, provided written informed consent to the inclusion of material pertaining to themselves, and they acknowledged that they could not be identified via the paper. All experimental protocols of this research project were approved by the Ethics Committee of Tamagawa University (Tokyo, Japan; approval no. TRE18-030).

### Participants

In this study, data from previous large-scale research projects conducted (Neuro-Psychological and Socio-Institutional Foundations of Pro-Social Behavior, http://www.human-sociality.net/english/) were reanalyzed. Although the research project was completed in 2018, it was used for the analysis in the current study for the effective use of data. The details of the research project are summarized in S1 Fig. Approximately 500 participants, between the ages of 20 and 50, participated in the experiment by taking part in multiple economic games, cognitive tasks, and psychological scales. Magnetic resonance imaging data, hormonal data, and genetic data were acquired. Written informed consent was obtained from all participants prior to enrollment in the study. All experimental protocols of this research project were approved by the Ethics Committee of Tamagawa University (Tokyo, Japan; approval no. TRE18-030).

The participants completed the tasks in individual booths to ensure their anonymity. General trust and caution scales were measured in the first wave (n = 564; May 2012–July 2012), third wave (n = 489, April 2013–June 2013), and sixth wave (n = 470; May 2014–July 2014). Saliva was collected from 205 participants who participated in the ninth wave (n = 290; November 2016–March 2018). Measurements using the general trust scale was also collected in the tenth wave (n = 307, July 2018–November 2018), but since caution was not collected in that wave, it was removed from the analysis.

We analyzed 197 participants (men = 98, women = 99, mean age = 41.7 years, standard deviation [SD] for age = 10.4 years) who completed the general trust and caution scales three times (first, third, and sixth waves) and provided their saliva samples (S1 Dataset). Although data from this dataset have already been reported in several studies [23–31], this study appears to be the first to investigate the association between salivary oxytocin levels and general trust and caution.

### General trust and caution

We measured the trust level in individuals by using the General Trust Scale and measured the caution level by using the Caution Scale [21]. These trust and caution scales use seven-point Likert scale (ranging from 1 for “strongly disagree” to 7 for “strongly agree”), and includes five items that measure the participants’ general trust (e.g., “Do you think most people can be trusted?”) and five items for caution (e.g., “Do you think you cannot be too careful in dealing with people?”). Participants answered the Trust and Caution Scale three times on different days. To reduce measurement errors, the scores of each participant were normalized and averaged before their use in further analyses. In addition to Yamagishi’s general trust and caution scales, the database also includes trust behavior in trust games and general trust scale use in General Social and World Value Surveys. However, the two latter measures could not distinguish between general trust and caution, which constitute trust as hypothesized in this study. Therefore, in this study, only Yamagishi’s general trust and caution scales were used to examine the relationship with salivary oxytocin level.

### Salivary oxytocin levels

To assess participants’ endogenous oxytocin levels, we measured oxytocin concentrations in saliva. We collected saliva from participants at rest, with the Saliva Collection Aid (Salimetrics, LLC., Carlsbad, CA, USA), using the passive drool method at the beginning of the experiment in the ninth wave of the project. Participants were asked to provide at least 1.2 mL of saliva. Samples were collected in cryovials and immediately stored at -80°C. Before the assay, we freeze-dried 1 mL of saliva with a freeze dryer (FD-1000; Tokyo Rikakikai Co. Ltd., Tokyo, Japan) for approximately 16 hours overnight. The freeze-dried sample was dissolved by adding an assay buffer four times the volume of the saliva sample. An enzyme-linked immunosorbent assay was conducted using a commercially available oxytocin kit (Enzo Life Sciences, Inc., Farmingdale, NY, USA). The assay was conducted in duplicate, and the concentration was calculated by using a microplate reader (Sunrise Rainbow RC-R; TECAN Group, Ltd., Zürich, Switzerland), based on relevant standard curves. The intra- and inter-assay coefficients of variation were 6.7% and 8.4%, respectively.

We measured the oxytocin level in saliva, following the manufacturer’s protocol, except for the extraction step. The extraction step was conducted by using a novel filtration method to remove interfering proteins to avoid erroneously tagging other molecules as oxytocin. This step is considered necessary, especially for blood samples [32]. In a previous study [33], the unextracted plasma oxytocin level was much higher than the extracted oxytocin level and no correlation existed between these two oxytocin levels, when measured by different procedures. However, Carter [34] pointed out the possibility that oxytocin measurement in blood involving the extraction step may result in the underestimation of the oxytocin level because oxytocin is filtered by its binding to other molecules in the plasma. A recent study found a high correlation between extracted and unextracted oxytocin levels in dogs, which demonstrated the validation of measuring without extraction in saliva methodologically [35]. Moreover, measurement using the extraction step is capable of inducing a high intra-assay coefficient of variation and unreliable results [35,36]. Therefore, some recent studies [36–38] have used unextracted oxytocin samples to measure the oxytocin level and have examined the relationship between oxytocin level and human sociality.

### Statistical analysis

One hundred and ninety-seven participants (men = 98, women = 99, mean age = 41.7 years, SD for age = 10.4 years) with data on three parameters, i.e., general trust, caution, and oxytocin, were included in the analysis. This study evaluated two indicators: general trust and caution. Therefore, the Bonferroni-corrected *p*-value (i.e., *p* = .025) was the significance level. Since salivary oxytocin concentrations were not normally distributed (*W* = 0.84, *p* < .0001, S2 Fig), nonparametric analyses (Spearman’s rank correlation coefficient) were performed for the association between oxytocin and other variables. Because this study reanalyzed data already in the database, a preliminary sample design was not possible. Therefore, using G*Power 3.1 [39], a sensitivity power analysis based on number of participants = 197 and power = 0.8 showed that this study could theoretically detect an effect size of 0.197 or higher.

## Results

The mean salivary oxytocin level was (expressed as the mean ± SD) 49.2 ± 24.6 pg/mL. The levels of salivary oxytocin were not associated with age (*r*_*s*(195)_ = -.10, *p* = .150, S3 Fig) or sex [*Mdn*_male_ = 42.835, *Mdn*_female_ = 42.38, Mann-Whitney’s U test:*U*(*N*_male_ = 98, *N*_female_ = 99,) = 4418.5, *z* = 1.08, *p* = .280, S4 Fig].

General trust was positively correlated with age (*r*_(195)_ = .21, *p* = .003), but caution was not associated with age (*r*_(195)_ = -.09, *p* = .214). No sex differences in general trust (*t*_(195)_ = 0.97, *p* = .334) and caution (*t*_(195)_ = 0.74, *p* = .462) were found. The three measurements for general trust and caution, and the correlations between the measurements, are shown in S1 Table. High correlations were observed between measurements.

**Fig 1:**
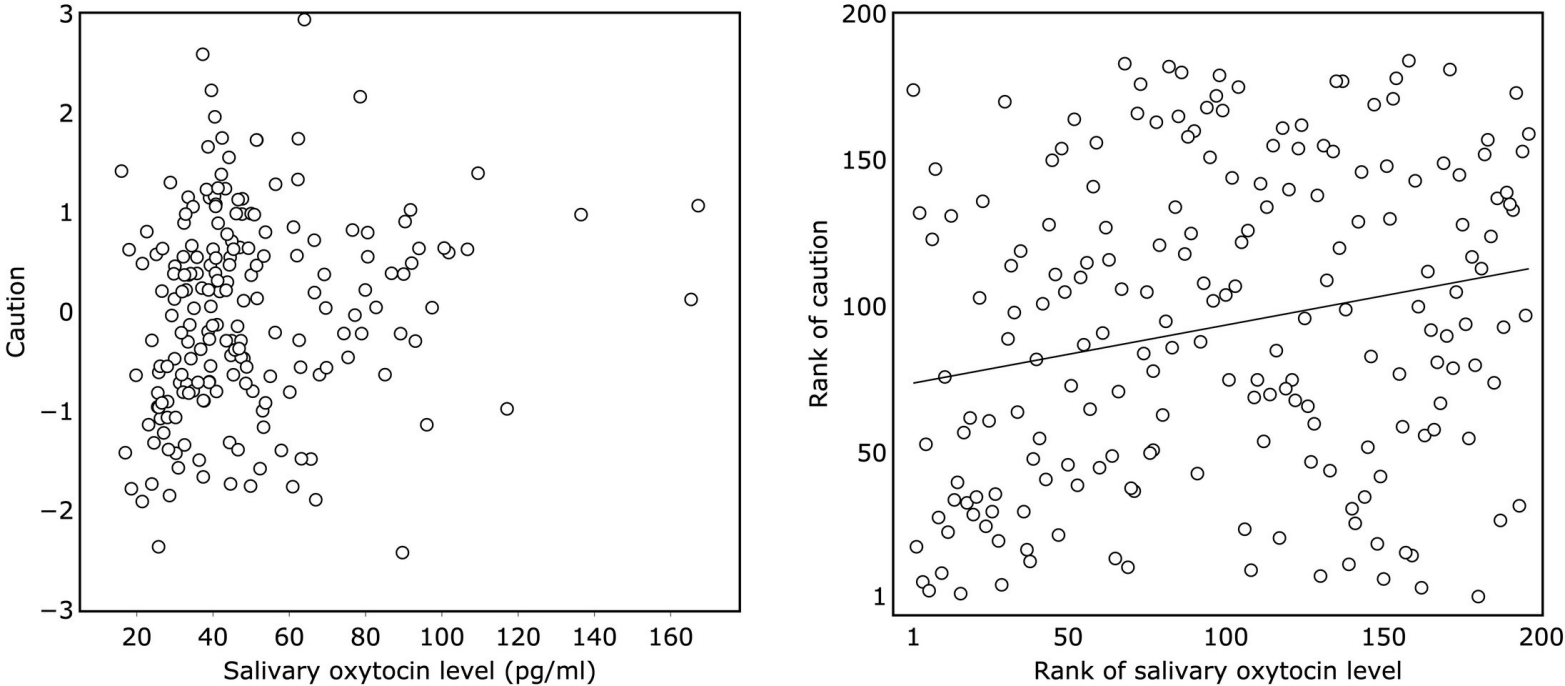
The association between salivary oxytocin level and caution. (A) scatter plot of salivary oxytocin level and caution. (B) scatter plot of rank of salivary oxytocin and rank of caution.

**Fig 2:**
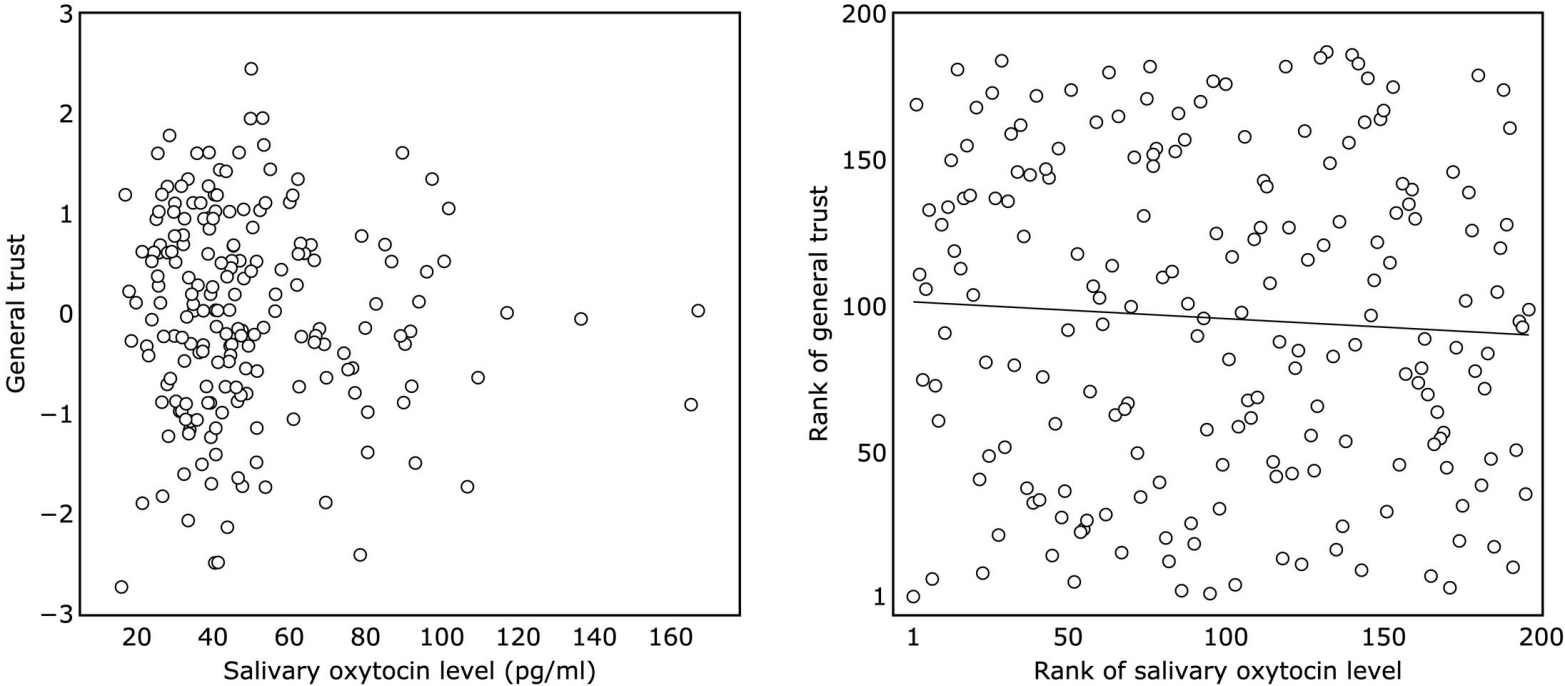
The association between salivary oxytocin level and general trust. (A) scatter plot of salivary oxytocin level and general trust. (B) scatter plot of rank of salivary oxytocin and rank of general trust.

People with higher salivary oxytocin levels tended to show a higher level of caution (*r*_*s*(195)_ = .21, *p* = .0025, Fig 1), but salivary oxytocin levels were not associated with general trust (*r*_*s*(195)_ = -.06, *p* = .395, Fig 2). The correlation between salivary oxytocin levels and caution was significant even after controlling for age and sex (*r*_*s*(195)_ = .20, *p* = .0048). Correlation analysis for caution was also conducted for each measurement on three different days, and all correlations were significant (1st: *r*_*s*(195)_ = .16, *p* = .024, S5 Fig; 2nd: *r*_*s*(195)_ = .17, *p* = .014, S6 Fig; 3rd: *r*_*s*(195)_ = .22, *p* = .0022, S7 Fig).

Next, we examined whether the results of the correlation differed by sex. In men, salivary oxytocin levels were positively correlated with caution at the 5% level (*r*_*s*(96)_ = .21, *p* = .040), but not with general trust (*r*_*s*(96)_ = .005, *p* = .962). The same pattern was observed in females (caution: *r*_*s*(97)_ = .21, *p* = .040, general trust: *r*_*s*(97)_ = -.13, *p* = .212).

## Discussion

In this study, we focused on two different aspects of trust that have not received much attention, namely general trust and caution, and investigated which of these two aspects of trust was regulated by oxytocin. This study found for the first time that salivary oxytocin was associated with the belief in caution against social uncertainty, rather than the belief in the trustworthiness of others.

The results of this study raise two important issues: the first issue is the finding that oxytocin is associated with caution rather than general trust. Previous studies have indicated that the intranasal administration of oxytocin could enhance trust behavior in a trust game [4,5,40]. However, in recent years, the effects of oxytocin on trust behavior have been questioned. Trust behavior in a trust game may be determined by various factors such as expectation of the trustworthiness of others and the tendency of betrayal aversion; and the weighting of each factor has individual differences [41,42]. One possibility is that oxytocin may influence trust behavior by altering the general trust level; for instance, intranasal oxytocin administration may increase the expectations regarding other people’s trustworthiness and kindness. Another explanation, based on caution, is that the intranasal administration of oxytocin may reduce one’s cautiousness and fear of betrayal. Our data exemplifies the second explanation. The study by Israel et al. [22] showed that oxytocin could affect the sensitivity in the perception of social deception, whereas it could not alter the perception of kindness in others. Our findings provide additional evidence that oxytocin cannot alter the perception of kindness in other people. Moreover, the hypothesis raised by Baumgartner et al. [4] that oxytocin increases trust behavior by reducing fear of betrayal and anxiety, as implied by decreased amygdala activity, is consistent with our result that salivary oxytocin levels were associated with caution. As shown in the current study, oxytocin functionally regulates caution; therefore, the administration of oxytocin may be effective in people with a strong weighting of betrayal avoidance tendency. A previous study [8] has indeed shown that the effect of oxytocin on trust-behavior depends on trust-attitude. Future studies need to consider the relationship between oxytocin and trust, while taking into account the belief of caution.

The second issue, as shown in our study, is concerned with the positive correlation between salivary oxytocin and caution level. We collected saliva without any task, which meant that the salivary oxytocin levels in this study were the basal endogenous oxytocin levels. Previous research has examined the relationship between basal oxytocin level and human sociality. Higher endogenous oxytocin levels were associated with supportive communication [43] and positive parenting styles [44,45]. However, higher oxytocin levels were also correlated with perceptions of interpersonal distress [17], life dissatisfaction [46], and major depression [47]. Oxytocin release because of external stimuli is considered to have an important role in reducing anxiety-like behavior in animals [12,13], although many studies have found that high basal plasma oxytocin levels without any stimuli were associated with increased anxiety in people with social anxiety disorder [46,48], as well as in healthy subjects [49,50]. Previous studies described an inverse relationship between endogenous neuromodulator concentration and function [51] or the density [52] of the corresponding receptor. An inverse relationship may also exist between the oxytocin level and oxytocin receptor function. Hoge et al. [46] found a positive relationship between plasma oxytocin level and anxiety in patients with social anxiety disorder. They proposed the hypothesis that patients with social deficits due to anxiety or autism may have higher levels of oxytocin, possibly as a compensatory mechanism in the face of malfunctioning oxytocin receptors. This finding indicates that high peripheral oxytocin levels may imply impaired oxytocin functions in anxiety attenuation. We suggest that people with high endogenous oxytocin levels have difficulties in dealing with anxiety in interpersonal interactions, which sensitizes their perception of the social environment and naturally increases the caution level when dealing with others.

Several limitations of the present study should be taken into consideration. First, although we found that the salivary oxytocin level could partly predict the trust level, explaining how salivary oxytocin can influence trust or how trust relates to salivary oxytocin levels is difficult. Second, this study analyzed data from a large database of prosociality, which approach using a large-scale database has an advantage in that a large amount of data can be used, but it also has a disadvantage in that the time for acquiring each dataset is different. In this study, to reduce the measurement error of general trust and caution, we used an index that averaged three datasets acquired at different times. However, the time of saliva collection was different among these times. In future studies, investigating the association between salivary oxytocin and general trust and caution on the same day will be necessary. In addition, though the validity of salivary oxytocin level measurement in this study has been demonstrated in a previous study [53], some studies have shown that the endogenous oxytocin level differed, depending on the measurement method that was used such as radioimmunoassay and enzyme-linked immunosorbent assay, or by using different measuring protocols [32,33]. Therefore, measuring salivary oxytocin levels by using different methods could help to replicate our study findings and increase the robustness. Moreover, the present study focused on the basal level of salivary oxytocin, but the reliability of the basal level of peripheral oxytocin remains in debate [54]. Thus, replication studies with other methodologies (e.g., administering exogenous oxytocin or investigating oxytocin receptor gene) are warranted to test the robustness of the present findings. In the future, we will consider examining the effects of exogenous oxytocin or changes in endogenous oxytocin levels from the baseline levels. This investigation may help scientists better understand the association between the salivary oxytocin level and attitudinal trust. Furthermore, we suggest that the salivary oxytocin level can be used as a biomarker for human prosociality and that examining the connection between salivary oxytocin and additional prosocial behaviors (e.g., reciprocity, cooperation) that are also associated with oxytocin is necessary [11,55].

## Conclusion

Our study demonstrated that salivary oxytocin level can act as a biomarker that partially predicts one’s trust level, especially based on caution. According to Yamagishi’s trust theory [20], trust consists of two independent components: general trust and caution. We believe that this study is the first to investigate that salivary oxytocin levels are only associated with caution, which is a component of trust. We believe that such an attempt to clarify the biological mechanism behind the social science theory on which it is based is important not only in life sciences but also in social sciences. In conclusion, Oxytocin has been called the "trust" hormone, but our study showed that oxytocin should be called the "assurance" hormone rather than the "trust" hormone.

## Supporting information

S1 Fig Overview of the whole research project. (DOCX)

S2 Fig Distribution of salivary oxytocin levels. (DOCX)

S3 Fig Scatter plot of salivary oxytocin level and age. (DOCX)

S4 Fig Mean salivary oxytocin levels by sex. (DOCX)

S5 Fig Scatter plot of salivary oxytocin level and caution measured for the first time. (DOCX)

S6 Fig Scatter plot of salivary oxytocin level and caution measured for the second time. (DOCX)

S7 Fig Scatter plot of salivary oxytocin level and caution measured for the third time. (DOCX)

S1 Table Mean levels and correlation coefficients for general trust and caution. (DOCX)

S1 Dataset Data used for the analysis reported in the article. (DOCX)
