# Supplementary figures and images for: Is oxytocin a trust hormone? Salivary oxytocin is associated with caution but not with general trust
Source: PLoS One. 2022 May 6;17(5):e0267988. doi: 10.1371/journal.pone.0267988 (PMC9075672; doi:10.1371/journal.pone.0267988)

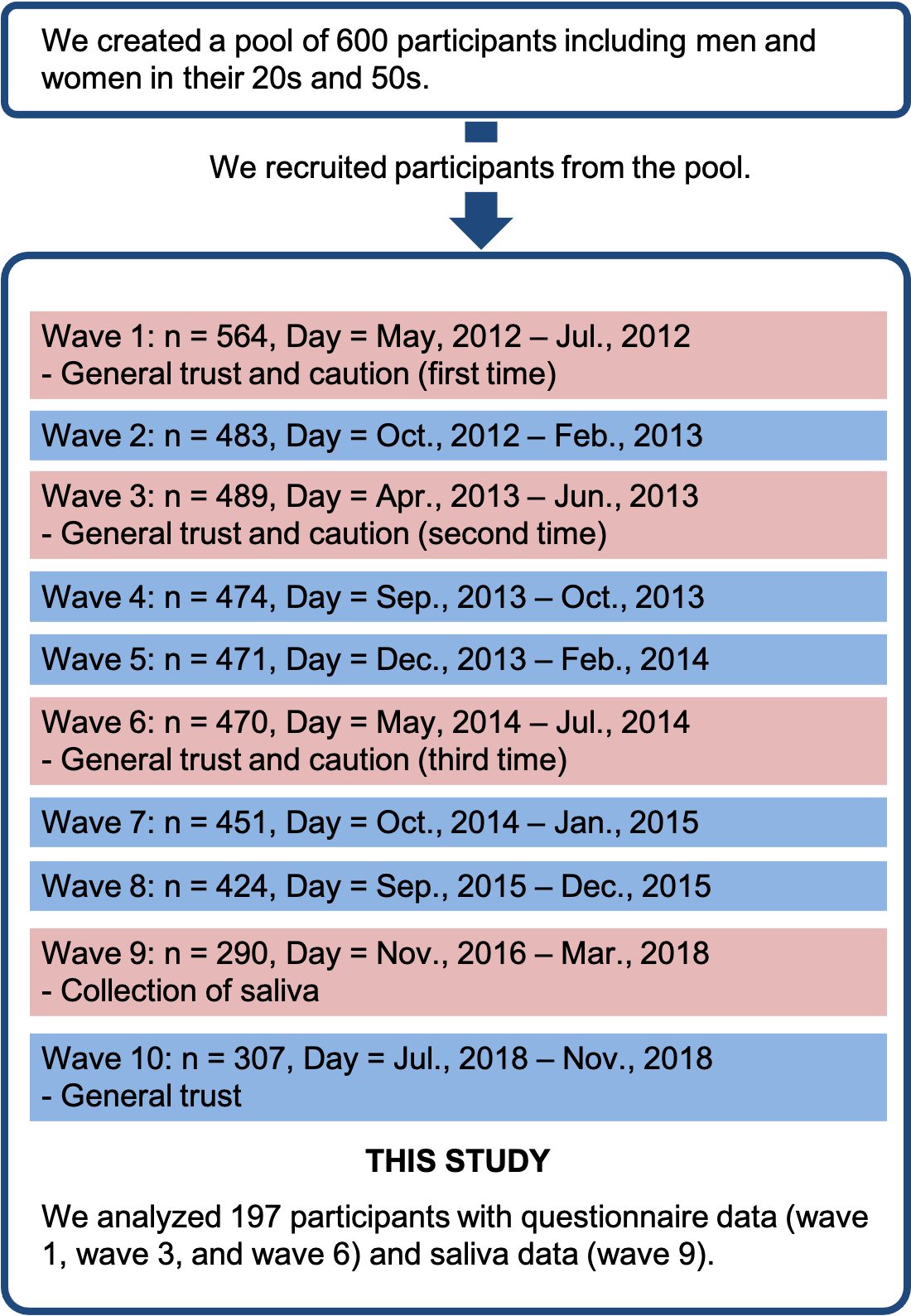


S1 Figure. Overview of the whole research project

Supplement: S1 Fig — (DOCX) [file pone.0267988.s001.docx]

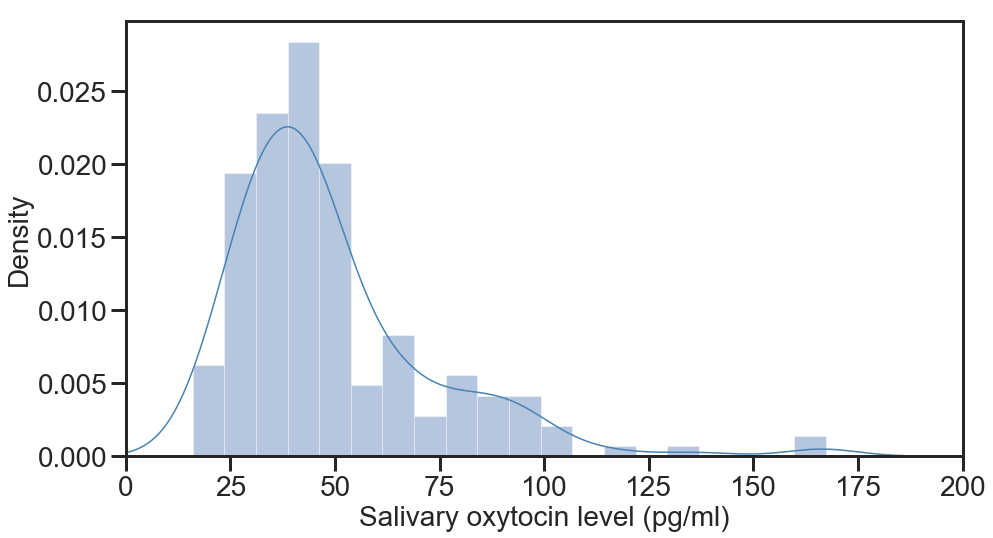


S2 Figure. Distribution of salivary oxytocin levels

Supplement: S2 Fig — (DOCX) [file pone.0267988.s002.docx]

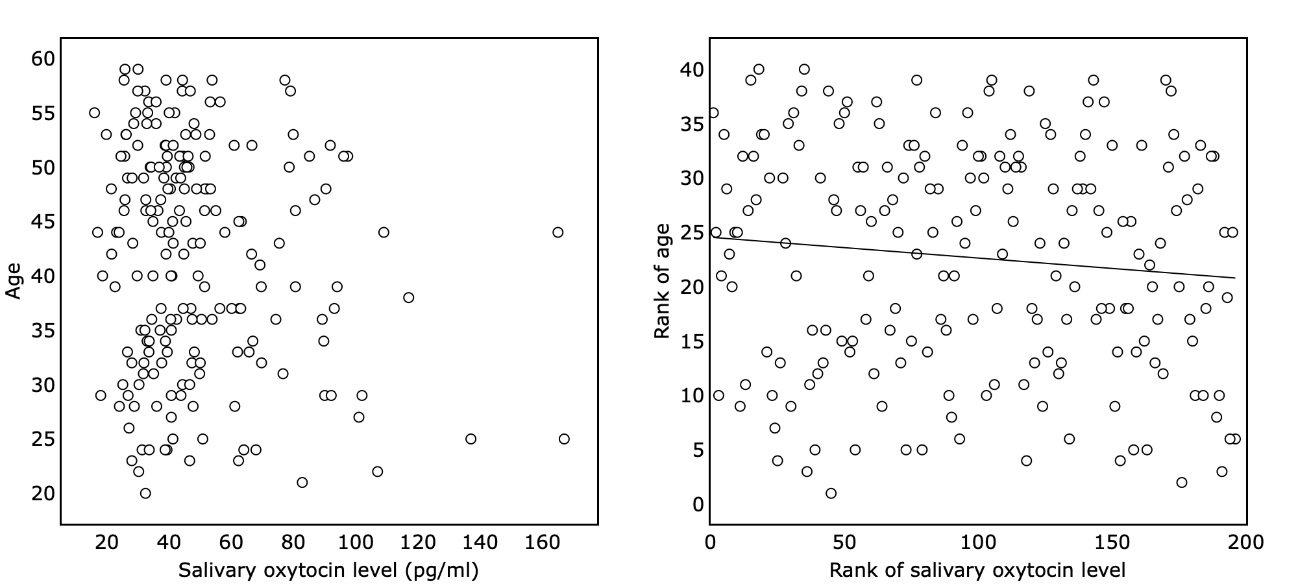


S3 Figure. Scatter plot of salivary oxytocin level and age

Supplement: S3 Fig — (DOCX) [file pone.0267988.s003.docx]

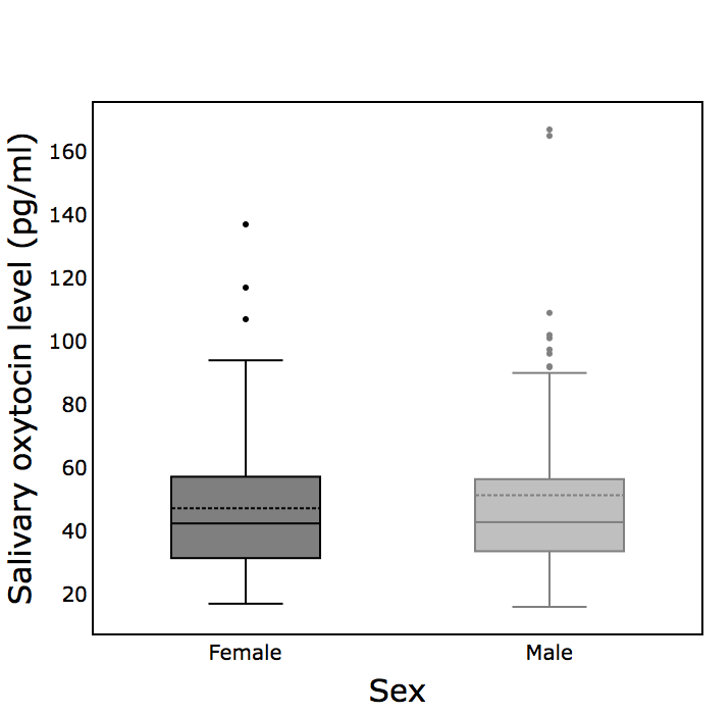


S4 Figure. Mean salivary oxytocin levels by sex

Supplement: S4 Fig — (DOCX) [file pone.0267988.s004.docx]

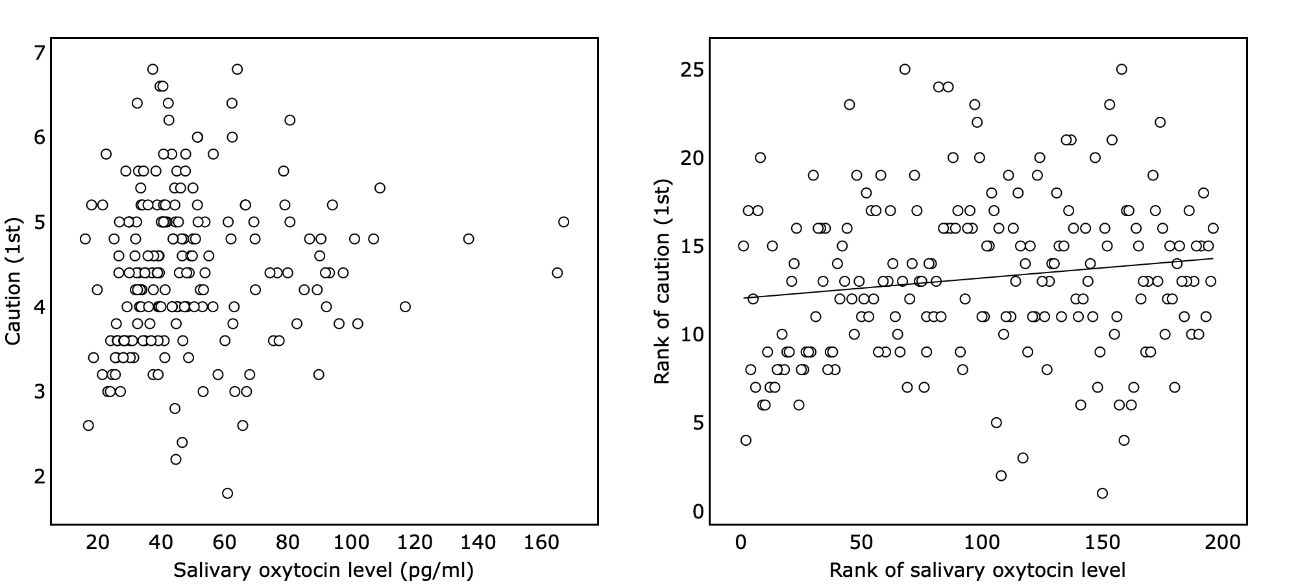


S5 Figure. Scatter plot of salivary oxytocin level and caution measured for the first time

Supplement: S5 Fig — (DOCX) [file pone.0267988.s005.docx]

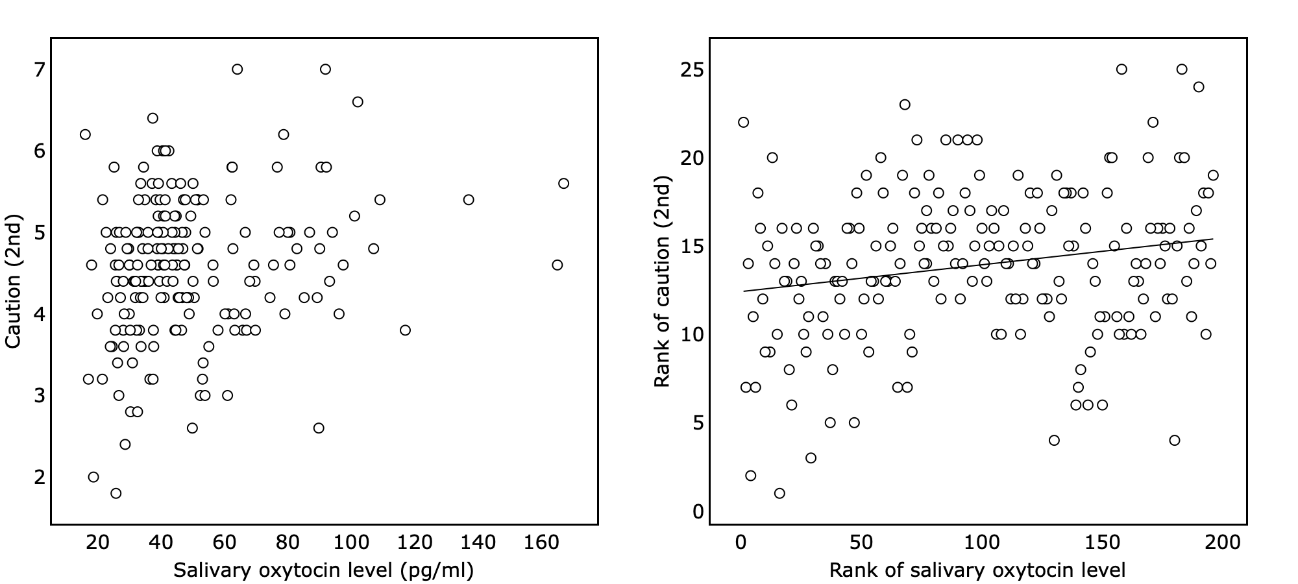
S6 Figure. Scatter plot of salivary oxytocin level and caution measured for the second time

Supplement: S6 Fig — (DOCX) [file pone.0267988.s006.docx]

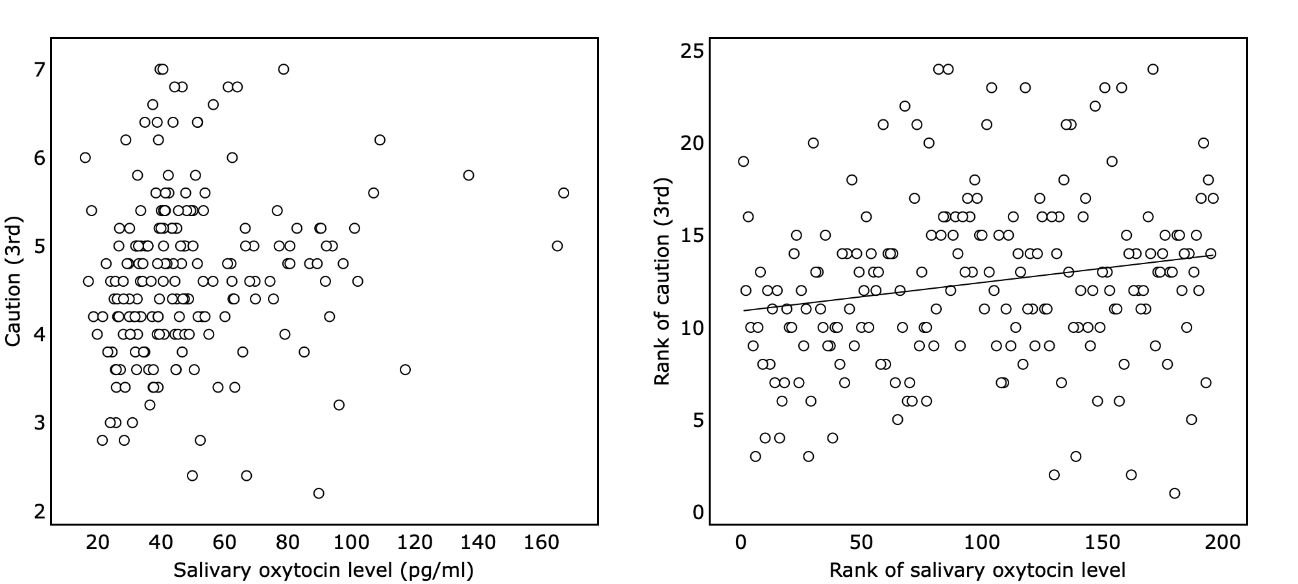


S7 Figure. Scatter plot of salivary oxytocin level and caution measured for the third time

Supplement: S7 Fig — (DOCX) [file pone.0267988.s007.docx]
