# Supplementary material for: Is oxytocin a trust hormone? Salivary oxytocin is associated with caution but not with general trust
Source: PLoS One. 2022 May 6;17(5):e0267988. doi: 10.1371/journal.pone.0267988 (PMC9075672; doi:10.1371/journal.pone.0267988)
Supplement: S1 Table — (DOCX) [file pone.0267988.s008.docx]

S1 Table. Mean levels and correlation coefficients for general trust and caution

| Variable | *M* | *SD* | 1 | | 2 | | 3 | | 4 | | 5 | |
| --- | --- | --- | --- | --- | --- | --- | --- | --- | --- | --- | --- | --- |
| 1. general trust ^a^ | 4.21 | 0.98 | - |  |  |  |  |  |  |  |  |  |
| 2. general trust ^b^ | 4.45 | 0.92 | .52 | ^**^ | - |  |  |  |  |  |  |  |
| 3. general trust ^c^ | 4.26 | 0.94 | .60 | ^**^ | .61 | ^**^ | - |  |  |  |  |  |
| 4. caution ^a^ | 4.43 | 0.92 | -.24 | ^**^ | -.27 | ^**^ | -.32 | ^**^ | - |  |  |  |
| 5. caution ^b^ | 4.58 | 0.87 | -.14 |  | -.33 | ^**^ | -.32 | ^**^ | .59 | ^**^ | - |  |
| 6. caution ^c^ | 4.68 | 0.94 | -.19 | ^**^ | -.26 | ^**^ | -.40 | ^**^ | .64 | ^**^ | .62 | ^**^ |

^a^ = data from wave 1, ^b^ = data from wave 3, ^c^ = data from wave 6, ***p* < .01.
