# Supplementary material for: Is oxytocin a trust hormone? Salivary oxytocin is associated with caution but not with general trust
Source: PLoS One. 2022 May 6;17(5):e0267988. doi: 10.1371/journal.pone.0267988 (PMC9075672; doi:10.1371/journal.pone.0267988)
Supplement: S1 Dataset — (DOCX) [file pone.0267988.s009.docx]

S1 Dataset. Data used for the analysis reported in the article

| ID | Age (y) | Sex (male = 1) | sOT level | GT (1st) | GT (2nd) | GT (3rd) | CA (1st) | CA (2nd) | CA (3rd) |
| --- | --- | --- | --- | --- | --- | --- | --- | --- | --- |
| 10056 | 46 | 0 | 36.37 | 3.4 | 3.8 | 4.8 | 3.8 | 3.2 | 3.2 |
| 10059 | 31 | 0 | 35 | 3.8 | 4.2 | 5 | 4.4 | 4.4 | 5 |
| 10068 | 35 | 0 | 37.09 | 4.2 | 2.2 | 3 | 4.4 | 5.6 | 4.2 |
| 10076 | 22 | 0 | 106.71 | 2.8 | 2.8 | 3.2 | 4.8 | 4.8 | 5.6 |
| 10082 | 28 | 1 | 47.7 | 2.6 | 3 | 3.2 | 5.8 | 5 | 5.6 |
| 10088 | 30 | 1 | 44.34 | 5.6 | 5.4 | 4.4 | 2.8 | 3.8 | 4 |
| 10091 | 20 | 1 | 32.39 | 1 | 4.2 | 3.8 | 6.4 | 5 | 4.4 |
| 10112 | 53 | 1 | 53.01 | 5.4 | 6.4 | 5.8 | 4 | 3.2 | 4.2 |
| 10117 | 37 | 0 | 47.02 | 4.4 | 4.8 | 5 | 4.8 | 5.4 | 5 |
| 10123 | 58 | 1 | 53.75 | 2.8 | 2.4 | 3.6 | 5 | 5 | 5.6 |
| 10125 | 24 | 0 | 31.25 | 3.4 | 3.8 | 3.4 | 3.4 | 4.4 | 4.2 |
| 10127 | 33 | 1 | 48.08 | 4.8 | 4.4 | 4.6 | 4 | 4.2 | 4.4 |
| 10130 | 37 | 0 | 44.48 | 4.6 | 3.8 | 3.8 | 5 | 4.8 | 5.2 |
| 10132 | 38 | 0 | 117.1 | 5 | 4 | 4 | 4 | 3.8 | 3.6 |
| 10138 | 29 | 1 | 89.98 | 3.4 | 4 | 3.4 | 4.6 | 4.8 | 5.2 |
| 10147 | 24 | 0 | 39.29 | 3.6 | 4 | 3.2 | 4.6 | 5 | 5.2 |
| 10159 | 59 | 1 | 30 | 5.4 | 5 | 5.2 | 5 | 4.6 | 5.2 |
| 10161 | 55 | 1 | 33.12 | 3 | 5.2 | 4.6 | 4 | 3.8 | 4.2 |
| 10165 | 30 | 0 | 30.27 | 2.6 | 4 | 4.2 | 3.6 | 2.8 | 4 |
| 10168 | 49 | 0 | 31.79 | 3.4 | 4 | 3.2 | 4.2 | 4.2 | 3.8 |
| 10170 | 53 | 0 | 45.33 | 5.6 | 4.8 | 4.2 | 4 | 4.2 | 4 |
| 10183 | 31 | 0 | 76.57 | 3.4 | 5 | 3.2 | 4.4 | 5.8 | 5.4 |
| 10189 | 40 | 1 | 18.58 | 2.8 | 6 | 3.4 | 3.4 | 2 | 4.2 |
| 10199 | 39 | 0 | 22.63 | 5 | 4.2 | 3 | 5.8 | 5 | 4.8 |
| 10217 | 33 | 1 | 39.43 | 3.6 | 3.2 | 3.2 | 4.4 | 5 | 4.4 |
| 10222 | 56 | 1 | 56.4 | 4.4 | 4.8 | 4.2 | 5.8 | 4.4 | 6.6 |
| 10223 | 28 | 1 | 61.09 | 3.4 | 4.2 | 2.8 | 5 | 4 | 6.8 |
| 10225 | 39 | 0 | 94 | 3.8 | 4.8 | 4.6 | 5.2 | 5 | 5 |
| 10230 | 44 | 1 | 109.49 | 3.4 | 3.8 | 4.2 | 5.4 | 5.4 | 6.2 |
| 10232 | 44 | 0 | 57.91 | 4.6 | 4.6 | 4.8 | 3.2 | 3.8 | 3.4 |
| 10235 | 44 | 0 | 16.99 | 5.4 | 5.2 | 5.2 | 2.6 | 3.2 | 4.6 |
| 10237 | 52 | 0 | 38.7 | 5 | 4.2 | 5.2 | 4.4 | 4.8 | 4 |
| 10244 | 33 | 0 | 62 | 4.2 | 5.4 | 4 | 4.8 | 5.4 | 4.8 |
| 10249 | 37 | 0 | 37.43 | 4 | 3.8 | 4.4 | 3.2 | 3.2 | 3.4 |
| 10252 | 28 | 1 | 23.96 | 4.4 | 4.6 | 3.8 | 3.6 | 4.8 | 4.6 |
| 10254 | 50 | 0 | 46.42 | 3 | 4.6 | 3.2 | 4.8 | 3.8 | 4.8 |
| 10260 | 40 | 0 | 40.9 | 3.6 | 4.8 | 4.2 | 3.6 | 4.2 | 4 |
| 10264 | 36 | 0 | 50.41 | 4.6 | 4.8 | 5.6 | 4 | 4.2 | 3.6 |
| 10271 | 51 | 1 | 97.44 | 5.8 | 4.8 | 5.6 | 4.4 | 4.6 | 4.8 |
| 10275 | 37 | 0 | 62.58 | 3.6 | 3.4 | 4.2 | 3.8 | 4.8 | 4.4 |
| 10277 | 23 | 1 | 46.58 | 3.2 | 3.8 | 2 | 4.6 | 5 | 6.8 |
| 10278 | 47 | 0 | 25.77 | 5 | 5.4 | 4 | 3.8 | 5 | 3.4 |
| 10290 | 52 | 1 | 29.87 | 4 | 4.4 | 4 | 5 | 4.6 | 4.4 |
| 10293 | 58 | 0 | 44.31 | 3.6 | 4.6 | 4.8 | 5.2 | 5 | 4.6 |
| 10300 | 48 | 0 | 44.93 | 4.4 | 4.8 | 5 | 5.6 | 4.6 | 5.2 |
| 10305 | 57 | 0 | 78.96 | 4.8 | 5 | 5 | 5.2 | 4 | 4 |
| 10312 | 43 | 0 | 47.61 | 5.2 | 3.4 | 4 | 5.6 | 5.4 | 5 |
| 10322 | 30 | 0 | 25.12 | 4.8 | 5 | 5.4 | 4.8 | 5.8 | 4.4 |
| 10326 | 33 | 0 | 65.69 | 5 | 4.8 | 4.8 | 2.6 | 3.8 | 3.8 |
| 10343 | 25 | 1 | 167.22 | 4 | 4.6 | 4.4 | 5 | 5.6 | 5.6 |
| 10344 | 51 | 1 | 96.05 | 1.6 | 6.2 | 6 | 3.8 | 4 | 3.2 |
| 10378 | 37 | 0 | 93.06 | 4 | 3.2 | 2.2 | 4.4 | 4.4 | 4.2 |
| 10381 | 36 | 0 | 42.38 | 3.8 | 3 | 3.8 | 6.2 | 6 | 5.6 |
| 10382 | 33 | 1 | 33.48 | 2.8 | 2.6 | 2.6 | 5.4 | 5.6 | 5.4 |
| 10386 | 29 | 0 | 43.76 | 2 | 3 | 2.8 | 4.8 | 4.8 | 4.8 |
| 10391 | 30 | 0 | 46.56 | 4.6 | 4.2 | 3.8 | 2.4 | 4.2 | 3.8 |
| 10393 | 50 | 1 | 39.09 | 5 | 5.8 | 4.4 | 4.6 | 5.6 | 6.2 |
| 10394 | 48 | 1 | 52.33 | 4.6 | 5.2 | 5.6 | 4.2 | 3 | 2.8 |
| 10398 | 46 | 0 | 55.01 | 5.6 | 5.8 | 5 | 4.6 | 3.6 | 4 |
| 10399 | 50 | 0 | 44.91 | 3.8 | 4 | 4.4 | 4 | 4.6 | 4.4 |
| 10404 | 29 | 1 | 101.8 | 4.6 | 7 | 3.8 | 3.8 | 6.6 | 4.6 |
| 10423 | 51 | 1 | 25.72 | 4.2 | 4.6 | 4.8 | 3.4 | 1.8 | 3 |
| 10432 | 43 | 0 | 50.02 | 5.8 | 6 | 7 | 4.8 | 4.4 | 5.4 |
| 10433 | 51 | 0 | 43.46 | 3 | 5 | 4.4 | 4 | 4.6 | 4.4 |
| 10434 | 48 | 0 | 40.39 | 5.2 | 5 | 5.6 | 5 | 6 | 5.4 |
| 10437 | 46 | 0 | 43.25 | 3.6 | 3.4 | 4.2 | 5.8 | 5.6 | 5.2 |
| 10451 | 54 | 1 | 28.59 | 5.4 | 5.8 | 6 | 3.6 | 2.4 | 3.4 |
| 10456 | 45 | 1 | 34.81 | 5 | 4.2 | 4 | 3.6 | 4.4 | 3.8 |
| 10458 | 42 | 1 | 44.7 | 4.2 | 5.6 | 4.2 | 3.8 | 5.2 | 3.6 |
| 10464 | 55 | 1 | 15.99 | 2 | 2 | 2.4 | 4.8 | 6.2 | 6 |
| 10473 | 50 | 0 | 33.89 | 4.2 | 3.2 | 2.8 | 4.2 | 4.4 | 4.8 |
| 10477 | 56 | 0 | 33.42 | 5.8 | 4.8 | 5.6 | 4.2 | 4.2 | 4.6 |
| 10481 | 35 | 0 | 30.9 | 4.8 | 4.8 | 5.2 | 3.6 | 3.4 | 3 |
| 10486 | 53 | 0 | 26.14 | 5 | 4.6 | 5 | 3.6 | 4.4 | 4.4 |
| 10488 | 23 | 0 | 62.26 | 6.4 | 5.6 | 4.2 | 6.4 | 5.8 | 4.6 |
| 10497 | 34 | 1 | 32.94 | 3.8 | 3.4 | 3.6 | 4.4 | 5 | 4.8 |
| 10501 | 51 | 1 | 51.55 | 4.2 | 3.2 | 4.2 | 5 | 4.8 | 4.2 |
| 10503 | 58 | 0 | 39.04 | 4.2 | 5.4 | 5.6 | 4 | 4.6 | 3.4 |
| 10506 | 50 | 0 | 78.59 | 2.8 | 1.8 | 2.6 | 5.6 | 6.2 | 7 |
| 10518 | 51 | 0 | 24.51 | 4.4 | 4.8 | 5.2 | 3.2 | 3.6 | 3.8 |
| 10525 | 49 | 1 | 38.27 | 3.8 | 4 | 3.4 | 5.6 | 5.4 | 5.6 |
| 10545 | 50 | 0 | 34.19 | 3.6 | 4.4 | 4.2 | 3.6 | 4.2 | 4.8 |
| 10547 | 57 | 0 | 32.21 | 4.8 | 5 | 4.8 | 5 | 5 | 5 |
| 10555 | 47 | 1 | 37.29 | 3.2 | 4.6 | 4.2 | 6.8 | 6.4 | 6.6 |
| 10567 | 48 | 0 | 21.44 | 4 | 4.8 | 5.6 | 3.2 | 3.2 | 2.8 |
| 10569 | 48 | 0 | 90.36 | 3.8 | 4.4 | 4 | 4.8 | 5.8 | 5.2 |
| 10577 | 51 | 0 | 39.35 | 4.6 | 5.2 | 3.6 | 4 | 4.4 | 4 |
| 10594 | 55 | 0 | 41.77 | 5.8 | 5.6 | 5 | 5 | 4.4 | 4.8 |
| 10597 | 37 | 1 | 60.12 | 5 | 5.4 | 5.2 | 3.6 | 4 | 4.2 |
| 10602 | 45 | 0 | 63.16 | 4 | 3.8 | 4.6 | 3 | 3.8 | 3.4 |
| 10603 | 39 | 0 | 69.56 | 2.4 | 3.2 | 2.8 | 4.8 | 4.4 | 4.6 |
| 10610 | 53 | 1 | 26.2 | 4.4 | 4.6 | 4.2 | 3.6 | 3.4 | 4.2 |
| 10619 | 48 | 1 | 39.57 | 2.2 | 4.8 | 1.8 | 6.6 | 5.4 | 7 |
| 10620 | 34 | 1 | 89.67 | 5.4 | 5.2 | 6.2 | 3.2 | 2.6 | 2.2 |
| 10623 | 46 | 1 | 25.49 | 3.8 | 5.2 | 4.8 | 3.2 | 4.6 | 3.6 |
| 10626 | 42 | 0 | 66.54 | 4.4 | 5.2 | 4.6 | 5.2 | 5 | 5.2 |
| 10629 | 39 | 1 | 80.57 | 3.6 | 3.2 | 3.8 | 5 | 5 | 5 |
| 10630 | 32 | 0 | 28.04 | 5.6 | 5.4 | 5 | 3.4 | 3.8 | 4 |
| 10631 | 53 | 0 | 48.6 | 3.2 | 4.2 | 4.2 | 3.4 | 4.2 | 4.4 |
| 10644 | 35 | 1 | 32.17 | 4.6 | 5.6 | 4.6 | 4.4 | 3.8 | 3.6 |
| 10651 | 41 | 1 | 69.17 | 3.8 | 4.2 | 4.2 | 5 | 4.6 | 5 |
| 10663 | 24 | 1 | 33.64 | 1.8 | 4 | 4.2 | 5.2 | 4.4 | 5 |
| 10667 | 52 | 0 | 38.95 | 5.6 | 5.8 | 5.4 | 3.2 | 4.6 | 4.2 |
| 10668 | 32 | 1 | 37.62 | 4.6 | 5.2 | 5.4 | 4.6 | 3.6 | 3.4 |
| 10675 | 49 | 0 | 26.6 | 3.4 | 4.4 | 3 | 4.6 | 4.6 | 5 |
| 10690 | 47 | 0 | 86.81 | 4.8 | 4.6 | 4.8 | 4.8 | 5 | 4.8 |
| 10696 | 44 | 0 | 23.1 | 3.5 | 5 | 3.4 | 3 | 4.2 | 3.8 |
| 10711 | 25 | 0 | 136.5 | 4.2 | 5 | 3.6 | 4.8 | 5.4 | 5.8 |
| 10712 | 39 | 1 | 51.41 | 5 | 5 | 4.2 | 5.2 | 4.8 | 4.8 |
| 10716 | 42 | 1 | 39.04 | 5.2 | 4.4 | 5.4 | 3.6 | 5.2 | 4.2 |
| 10718 | 40 | 0 | 34.75 | 5.2 | 5.2 | 5.2 | 4.4 | 5.4 | 6.4 |
| 10722 | 48 | 1 | 51.48 | 3.2 | 3.4 | 3.6 | 6 | 5.4 | 6.4 |
| 10735 | 40 | 1 | 49.29 | 4.4 | 3.6 | 4.2 | 4.6 | 5.2 | 5.4 |
| 10738 | 26 | 0 | 27.06 | 4.4 | 4.6 | 3.4 | 3 | 4.2 | 3.6 |
| 10744 | 37 | 1 | 56.28 | 4.2 | 4.4 | 4.4 | 4 | 4.6 | 4.6 |
| 10752 | 36 | 1 | 47.37 | 3.8 | 4.4 | 4.2 | 4 | 4.6 | 4 |
| 10754 | 24 | 0 | 38.69 | 3 | 3.2 | 4.6 | 5.2 | 6 | 6.4 |
| 10755 | 24 | 0 | 63.95 | 5.2 | 5 | 4.2 | 6.8 | 7 | 6.8 |
| 10757 | 51 | 0 | 44.59 | 4.6 | 3 | 4.4 | 2.2 | 3.8 | 3.6 |
| 10764 | 48 | 0 | 48.81 | 3 | 4 | 4 | 4.4 | 4 | 4 |
| 10778 | 36 | 1 | 53.77 | 5.2 | 5.2 | 5.2 | 4.4 | 3 | 4.2 |
| 10780 | 24 | 1 | 67.91 | 4.8 | 4.4 | 3.4 | 3.2 | 4.4 | 4.6 |
| 10781 | 57 | 0 | 44.18 | 4.2 | 4.4 | 3.2 | 5.4 | 5.2 | 6.8 |
| 10791 | 42 | 0 | 21.48 | 2.6 | 2.8 | 3 | 5.2 | 5.4 | 4.2 |
| 10808 | 46 | 1 | 32.52 | 5 | 5.8 | 4.4 | 3.8 | 2.8 | 4 |
| 10810 | 54 | 0 | 35.8 | 5.8 | 5.4 | 5.6 | 4.6 | 5 | 5 |
| 10819 | 36 | 1 | 40.47 | 3.2 | 2.8 | 1 | 6.6 | 4.8 | 7 |
| 10821 | 49 | 0 | 42.17 | 5.6 | 4.4 | 4.2 | 6.4 | 4.8 | 5.8 |
| 10835 | 51 | 1 | 85.09 | 4.8 | 4.8 | 5 | 4.2 | 4.2 | 3.8 |
| 10838 | 34 | 1 | 33.63 | 4.6 | 5 | 4.2 | 4 | 3.6 | 4.2 |
| 10847 | 51 | 0 | 43.42 | 6 | 4.4 | 6 | 4 | 5 | 5.2 |
| 10848 | 43 | 0 | 75.41 | 3.6 | 3.8 | 4.2 | 3.6 | 4.6 | 4.4 |
| 10849 | 57 | 1 | 29.93 | 4.8 | 5 | 5 | 4.4 | 4 | 4.2 |
| 10850 | 32 | 0 | 47.32 | 3.8 | 3.6 | 3.6 | 4 | 4.6 | 4.4 |
| 10854 | 40 | 1 | 40.63 | 5.2 | 5.2 | 5 | 5 | 5 | 4.6 |
| 10863 | 50 | 0 | 45.72 | 4.4 | 4.6 | 4.4 | 4.4 | 4.2 | 4.2 |
| 10868 | 22 | 0 | 30.19 | 5 | 4.4 | 4.8 | 3.4 | 3.8 | 4 |
| 20932 | 55 | 1 | 40.01 | 4.8 | 5.6 | 4.8 | 5 | 4.8 | 5.4 |
| 20936 | 37 | 0 | 62.95 | 4.2 | 5 | 5.4 | 4 | 4 | 4.4 |
| 20944 | 57 | 1 | 46.83 | 5.6 | 5.8 | 5.4 | 3.6 | 4.8 | 4.4 |
| 20954 | 59 | 0 | 25.79 | 5.2 | 4.8 | 5.4 | 3.4 | 4.4 | 3.6 |
| 20982 | 27 | 1 | 40.74 | 3.8 | 3 | 2.8 | 5.2 | 6 | 5 |
| 20992 | 32 | 1 | 49.99 | 5.2 | 4 | 4.8 | 5.4 | 5.6 | 5 |
| 21013 | 34 | 1 | 66.94 | 3.8 | 4.4 | 4.2 | 3 | 3.8 | 2.4 |
| 21049 | 53 | 0 | 19.81 | 4.4 | 4.8 | 4 | 4.2 | 4 | 4 |
| 21050 | 35 | 1 | 40.68 | 4 | 5 | 4 | 5.8 | 5.2 | 5.2 |
| 21055 | 56 | 1 | 53.24 | 3.8 | 4.2 | 4.6 | 3 | 3.4 | 4.6 |
| 21057 | 53 | 1 | 79.92 | 4 | 4 | 4.6 | 4.4 | 5 | 4.8 |
| 21070 | 44 | 1 | 165.36 | 4.2 | 3.2 | 3.4 | 4.4 | 4.6 | 5 |
| 21076 | 46 | 1 | 34.1 | 3.8 | 2.8 | 3.8 | 5.2 | 4.8 | 4.6 |
| 21090 | 58 | 0 | 77.2 | 3.2 | 5 | 2.8 | 3.6 | 5 | 5 |
| 21100 | 25 | 0 | 41.2 | 3.8 | 4.4 | 4.8 | 4.2 | 5.4 | 4.8 |
| 21125 | 54 | 1 | 48.02 | 4.4 | 5.8 | 5.2 | 4.4 | 4.2 | 5.4 |
| 21129 | 36 | 1 | 74.31 | 4 | 4 | 4 | 4.4 | 4.2 | 4.6 |
| 21135 | 49 | 1 | 28.08 | 5 | 5.4 | 4 | 3.6 | 3.6 | 4.4 |
| 21150 | 43 | 1 | 28.31 | 3.2 | 3.6 | 3.2 | 3.6 | 4 | 2.8 |
| 21159 | 45 | 1 | 45.38 | 5.2 | 4.8 | 4.6 | 5 | 4.8 | 5.4 |
| 21161 | 31 | 1 | 31.7 | 5.4 | 5.4 | 5.2 | 4.6 | 4.4 | 4.2 |
| 21230 | 52 | 1 | 66.59 | 3 | 5 | 4.2 | 5.2 | 4 | 5 |
| 21260 | 44 | 1 | 23.94 | 4.8 | 4.8 | 4.6 | 3 | 3.6 | 3 |
| 21269 | 56 | 1 | 35.79 | 3.6 | 3.8 | 3 | 5.2 | 4.8 | 5 |
| 21273 | 31 | 1 | 49.84 | 5.2 | 5.6 | 6.8 | 4.6 | 2.6 | 2.4 |
| 21278 | 32 | 0 | 69.74 | 3.6 | 4 | 3.8 | 4.2 | 3.8 | 4.4 |
| 21281 | 40 | 0 | 29.72 | 5.8 | 5.4 | 4.2 | 5 | 4.8 | 4.8 |
| 21289 | 58 | 1 | 25.52 | 6 | 5.6 | 5.2 | 3.4 | 3.8 | 4.6 |
| 21298 | 29 | 0 | 26.76 | 3.6 | 3 | 2 | 5 | 5 | 5.2 |
| 21331 | 27 | 1 | 100.61 | 4.8 | 4.8 | 4.6 | 4.8 | 5.2 | 5.2 |
| 21351 | 28 | 1 | 36.04 | 4.2 | 5.2 | 4.2 | 4 | 4.4 | 3.6 |
| 21360 | 46 | 1 | 51.4 | 3.4 | 3 | 3 | 6 | 5.4 | 6.4 |
| 21381 | 47 | 1 | 32.5 | 4 | 4.6 | 3.2 | 4.2 | 4.6 | 5.8 |
| 21389 | 34 | 0 | 38.81 | 5.2 | 5.2 | 5.6 | 4.4 | 5 | 4.8 |
| 21391 | 52 | 1 | 91.78 | 5.2 | 2.6 | 4.8 | 4.4 | 7 | 4.6 |
| 21394 | 33 | 0 | 26.59 | 5.2 | 5.2 | 5.4 | 4.4 | 3 | 4.2 |
| 21402 | 52 | 1 | 41.3 | 4.2 | 3.6 | 4 | 5 | 6 | 5.6 |
| 21420 | 51 | 1 | 46.08 | 4 | 3.4 | 3.8 | 5.4 | 5.6 | 5 |
| 21424 | 23 | 0 | 27.98 | 2.6 | 4.2 | 4.4 | 3.4 | 4.4 | 4.6 |
| 21444 | 29 | 1 | 40.73 | 3.4 | 3.6 | 3.2 | 5 | 4.6 | 5.4 |
| 21472 | 52 | 0 | 60.89 | 5.6 | 5 | 5.2 | 1.8 | 3 | 4.8 |
| 21473 | 29 | 1 | 92.08 | 3.4 | 3.6 | 4.2 | 4 | 5.8 | 5 |
| 21500 | 44 | 1 | 37.46 | 4.2 | 4.8 | 4 | 4.2 | 3.8 | 3.6 |
| 21516 | 43 | 1 | 41.33 | 2.4 | 2 | 2.6 | 5.2 | 5.2 | 5.4 |
| 21520 | 25 | 0 | 50.82 | 3.6 | 5.2 | 3.6 | 4.8 | 5.4 | 5.8 |
| 21549 | 29 | 0 | 17.98 | 3.2 | 5.8 | 4.4 | 5.2 | 4.6 | 5.4 |
| 21552 | 36 | 0 | 89.18 | 3.8 | 4.2 | 4.4 | 4.2 | 4.2 | 4.8 |
| 21555 | 32 | 1 | 31.9 | 3.75 | 4.6 | 4 | 4.8 | 4.4 | 5 |
| 21556 | 21 | 1 | 82.75 | 4.8 | 4.2 | 4.2 | 3.8 | 4.8 | 5.2 |
| 21571 | 54 | 1 | 32.83 | 3.2 | 3.6 | 3.6 | 5.6 | 5.4 | 5 |
| 21577 | 50 | 1 | 36.76 | 5.4 | 5.4 | 4.8 | 3.6 | 4.6 | 4.6 |
| 21594 | 45 | 1 | 62.37 | 5.2 | 4.4 | 4.8 | 6 | 5.8 | 6 |
| 21617 | 45 | 0 | 41.05 | 5.6 | 5.2 | 5 | 3.4 | 4.6 | 5.4 |
| 21634 | 48 | 1 | 53.32 | 6 | 5.6 | 5.4 | 4.2 | 5.4 | 5.4 |
| 21637 | 49 | 0 | 43.69 | 4 | 5 | 4.8 | 4.8 | 4.4 | 6.4 |
| 21654 | 28 | 0 | 28.81 | 4 | 3.8 | 3.6 | 5.6 | 5 | 6.2 |
| 21661 | 46 | 1 | 80.55 | 2.8 | 3.6 | 3.2 | 6.2 | 4.6 | 4.8 |
| 21688 | 44 | 1 | 39.87 | 4.8 | 4.4 | 4.4 | 4 | 4.2 | 5.2 |
| 21690 | 55 | 0 | 29.17 | 4.2 | 5.2 | 5 | 4 | 4.8 | 4.8 |
| 21729 | 36 | 1 | 34.42 | 4.2 | 4.6 | 4.6 | 5.6 | 5.8 | 3.8 |

sOT = salivary oxytocin, GT = general trust, CA = caution
